# Customizing implant emergence profile and provisional prostheses. Combination of the digital and the analog protocol. A Case Report

**DOI:** 10.4317/jced.61633

**Published:** 2024-06-01

**Authors:** George Kouveliotis, Ioannis Karoussis, Eirini Papamanoli, Theodoros Tasopoulos, Stefanos Kourtis, Mateus Rocha, Dayane Oliveira, Sherif Hosney, Panagiotis Zoidis

**Affiliations:** 1 DDS, CDT, MS. Clinical Assistant Professor and Director of Digital Center, Department of Restorative Dental Sciences, College of Dentistry, University of Florida, Gainesville, FL, USA; 2 DDS, MS, Dr Med Dent. Associate Professor, Department of Periodontology, Dental School, National and Kapodistrian University of Athens, Greece; 3 DDS. Dentist, Private Practice, Athens, Greece; 4 DDS, MS. Dentist, Private Practice, Athens, Greece; 5 DDS, Dr. Odont. Associate Professor, Department of Prosthodontics, Dental School, National and Kapodistrian University of Athens, Greece; 6 DDS, MS, PhD. Clinical Assistant Professor, Center for Dental Biomaterials, Department of Restorative Dental Sciences, College of Dentistry, University of Florida, Gainesville, FL, USA; 7 BDS, MS. Clinical Assistant Professor and Director of Graduate Prosthodontics, Department of Restorative Dental Sciences, College of Dentistry, University of Florida, Gainesville, FL, USA; 8 DDS, MS, PhD. Clinical Professor and Associate Dean for Clinical Affairs and Quality Assurance, Department of Restorative Dental Sciences, College of Dentistry, University of Florida, Gainesville, FL, USA

## Abstract

The emergence profile in implant restoration has an important contribution to the final esthetic result. Moreover, a properly shaped emergence profile enables well-performed dental hygiene and protects the implant-restoration complex from peri-implant infections. This report describes a clinical case that combines digital workflow and designing with a custom implant healing abutment system (Cervico). This system is utilized to customize the provisional restoration’s emergence profile with the conventional processing along with the digitally designed and milled provisional restoration to deliver an interim prosthesis after implant placement.

** Key words:**Cervico, Customized emergence profile, Digital Dentistry, Implant surgical guide, Implant provisionals.

## Introduction

Implant-supported restorations primarily have to serve function and esthetics. However, clinicians have to create prostheses that maintain soft tissue biological and mechanical stability and at the same time preserve longevity through patient’s easy oral hygiene (1). Hence, transitioning from an implant’s round neck to a natural prosthetic cervical anatomy is of great importance (2,3). Anatomical emergence profile is related not only to prostheses design but also to very detailed surgical planning and execution. Labiolingual implant position, distance to adjacent teeth, drilling depth and distance from the bone crest, inclination during positioning, and keratinized soft tissue thickness are some factors that determine the ideal emergence profile (4,5).

There are many different ways described to create an anatomical profile in implant-supported prostheses either with a provisional implant restoration where alterations of the morphology in the cervical area create a desired tissue design or by using customized abutments of the definite restoration (6,7). However, the techniques mentioned have the disadvantage of removing multiple times the restoration from its position which according to Abrahamsson *et al*. leads to an apical peri-implant tissue shifting and bone absorption (8-10).

Cervico System

**Figure 1:**
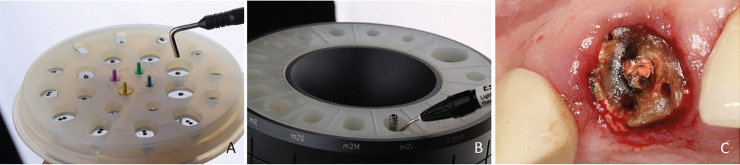
A. Cervico Guide. Indicators guide about teeth dimensions and final access hole position. B. Cervico Mold. Temporary abutments are placed into the silicone cells and screwed on the implant analogs below. Flowable resin is polymerized inside the cells before operation time and then disinfected in chlorhexidine solution to avoid cytotoxicity during intraoral placement. C. Initial clinical situation. Tooth #8 fractured horizontally.

**Figure 2:**
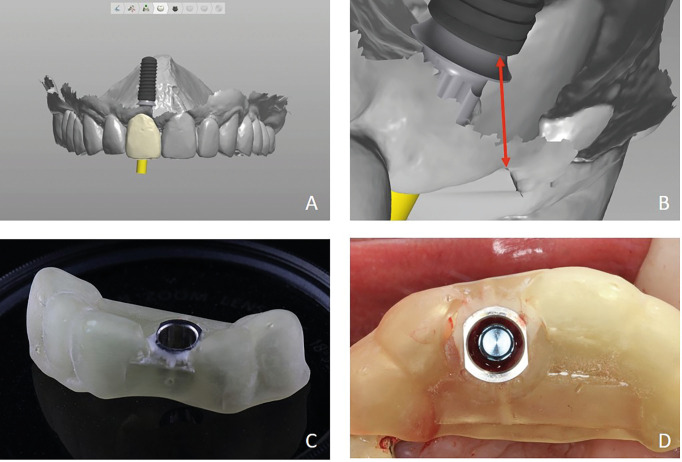
A. Digital implant position and design of the provisional restoration. B. Measurements between implant neck and soft tissue level. The cervical area of the provisional restoration is placed at this level. C. Surgical guide printed. D. Implant placement according to digital design.

Cervico (Innovato Holdings) is a system aiding the clinician to make a favorable position for the initial drilling during implantation (Cervico Guide) and it creates an anatomical emergence profile through the customization of healing abutments (Cervico Mold) (Figs. 1,2). The Cervico Guide gives information about teeth dimensions and guides the clinician on where the pilot drilling should be to have a screw-retained restoration. The Cervico Mold is a device filled with silicone cells that have the same dimensions as the indicators of the surgical part (Cervico Guide) and in addition to their ending, they have the cervical morphology of each teeth group (upper incisors, lower molars, etc.) and an access hole. Under this silicone index, a mock implant of the selected system brand is placed to screw on it a temporary abutment which will serve as a customized healing abutment after filling the silicone cell with flowable resin.

**Figure 3:**
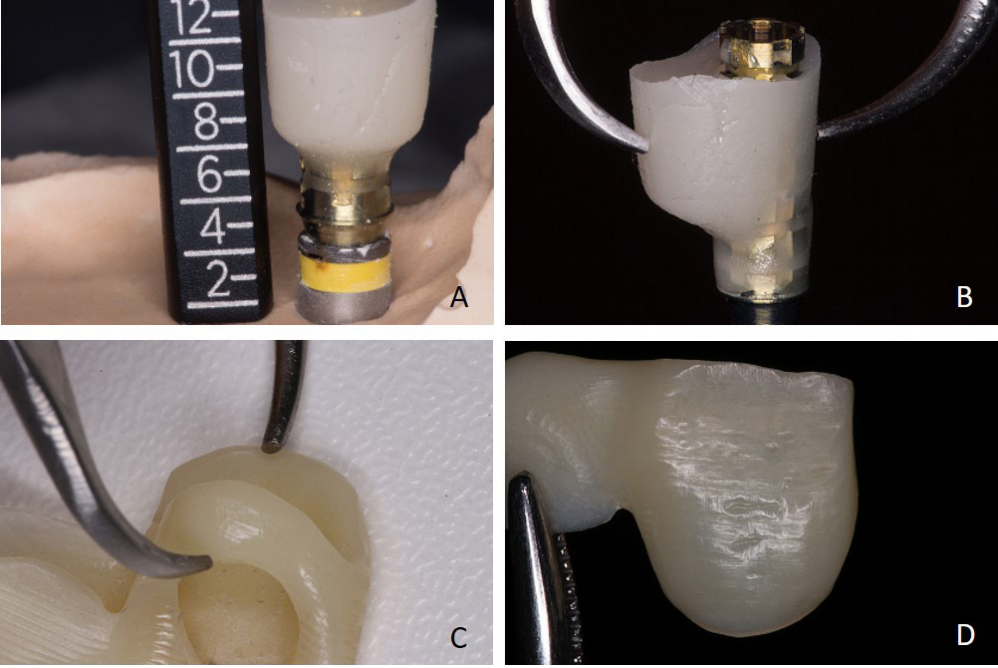
Cervico abutment dimensions are measured. A. Height. B. Width. C. Crown dimensions are measured. D. Incisal acrylic index stabilizing crown position.

In this dental technique, the Cervico system was combined with digital surgical protocol and digital designing of the temporary restoration during immediate loading. Initially, a female patient was referred for a fracture of the right upper central incisor (#8). Tooth extraction was decided following immediate implantation and loading with provisional prostheses for esthetic reasons (Fig. 3). The purpose of this dental technique was to create a customized provisional prosthesis in the esthetic zone respecting soft tissue morphology.

## Case Report

**Figure 4:**
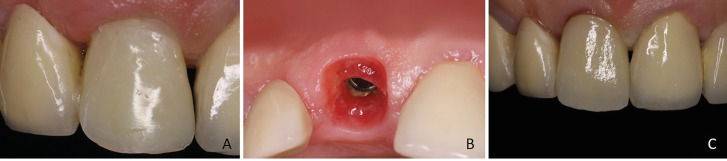
A. Provisional restoration immediately activated intraorally. B. Soft tissue structure after 12 weeks creating a natural emergence profile for the final restoration. C. Definitive restoration.

Cone Beam Computer Tomography (CBCT) radiograph was taken and the Digital Imaging and Communication in Medicine (DICOM) files were extracted. A digital impression was made (TRIOS 3, 3Shape AS). These Standard Tessellation Language (STL) files were combined with DICOM files in a specific software (Implant Studio, 3Shape AS) for digital implant positioning, designing of the provisional restoration, and the surgical guide (Fig. 4). Distances between the digitally positioned implant neck and the newly designed provisional crown cervical and incisal edge point. A Surgical guide was printed (Form 3, Formlabs) and a polymer provisional restoration (Telio CAD, Ivoclar Vivadent) was milled (MC X5, Dentsply Sirona).

A temporary abutment was positioned on the Cervico Mold and screwed in the anterior teeth position. Flowable resin was poured and a customized healing abutment resulted. Following this, the abutment was fastened to an implant analog and measured at all dimensions. These measurements were made also to the printed provisional restoration. The healing abutment resin was shaped into a temporary custom abutment having the proper dimensions for an intraoral activation after implant surgery.

Initial surgical incisions were made to avoid tooth fracture and to perform an atraumatic tooth extraction. Following the extraction, the surgical guide was positioned intraorally, its passive fit was checked and was not removed until the end of the operation. All surgical drillings were made according to the digital surgical protocol and the implant was placed (BLT, Straumann, Basel, Switzerland). Initial implant stability was measured at 50 N which allowed immediate implant provisionalization. The customized abutment was torqued at 35 N and provisional crowns were inserted passively using a positioning matrix seated on the left central incisor. After 12 weeks, according to the osseointegration protocol, the definitive prosthesis was delivered.

## Discussion

The emergence profile in implant restorations in the esthetic zone is well documented in the literature. Some authors divided it into two zones, the critical and the subcritical (10). The critical zone is below the soft tissue level at the implant-abutment connection complex which covers circumferentially the restoration. Buccally this zone defines the cervical level of the prostheses. The second zone, the subcritical, is located apically if there is adequate soft tissue height above the implant platform. Sancho- Puchades *et al*. mentioned that a concaved emergence profile retains more cement remnants in cement-retained restorations which is a high-risk factor for peri-implant inflammation (7).

Altering morphology in the cervical area in implant prosthodontics will lead to a more natural and pleasant tissue structure preserving interdental papilla and keratinized mucosa (10). The Cervico system permits the clinician to have a preoperative analysis of the implant position to deliver a screw-retained restoration. Moreover, fabricating customized healing abutments guides soft tissue healing to create and promote a natural emergence profile for the definite prostheses. Continuous intervention in this sensitive area and removal of the healing abutment and the temporary restoration according to Abrahamsson *et al*. will lead to apical removal of connective tissue and bone resorption (9).

Digital technology through its applications either designing or manufacturing has changed today’s Dentistry. Guided implantology facilitates the clinician to make a detailed examination of the operation area and place digitally the implant before surgery and that gives self-confidence during operation time (11). In addition, immediate loading is more predicTable and minimizes the healing period (12). Several systematic reviews refer that implants positioned using a digitally designed surgical guide had small position deviation (up to 1,6 mm) and angulation (6o) (13). The most common errors described are designing inaccuracies, problems during guide production time, sleeve misplacement, or guide fracture during operation. Maintenance of a 2 mm safety margin around vital structures during design could result in fewer problems during implant placement (14).

In this dental technique, digital technology was combined with analog techniques and materials to achieve an esthetic outcome. Knowledge of every detail of implant position related to adjacent tissues or teeth structures makes immediate loading not so stressful for the clinician and increases the accuracy and the predictability of the final outcome.

## Data Availability

The datasets used and/or analyzed during the current study are available from the corresponding author.
